# Effect of *Toxocara canis* infection on liver and lung microbial flora diversity and composition in dogs

**DOI:** 10.1051/parasite/2025011

**Published:** 2025-03-05

**Authors:** Na Wang, Soben Sieng, Tian Liang, Ping Chen, Jingyun Xu, Qian Han

**Affiliations:** 1 Laboratory of Tropical Veterinary Medicine and Vector Biology, School of Life and Health Sciences, Hainan Province Key Laboratory of One Health, Collaborative Innovation Center of One Health, Hainan University 58 Renmin Avenue Haikou 570228 Hainan China; 2 Hainan International One Health Institute, Hainan University 58 Renmin Avenue Haikou 570228 Hainan China; 3 Faculty of Veterinary Medicine, Royal University of Agriculture Dongkor District Phnom Penh 120501 Cambodia

**Keywords:** *Toxocara canis*, 16s rRNA high-throughput sequencing, Microbial flora, Liver, Lung

## Abstract

Toxocariasis is a zoonotic parasitic disease that is widely prevalent in the world. *Toxocara canis* adults are parasitic in the small intestinal tract of canids, and the larvae migrate to the liver and lungs before reaching the final destination. Our previous experiments have confirmed that *T. canis* infection could affect the composition of host intestinal microbial flora. In this experiment, we further analyze the potential effects of *T. canis* infection on host liver and lung microbial flora. Utilizing 16s rRNA high-throughput sequencing, coupled with various bioinformatics analysis techniques, our study revealed that *T. canis* infection significantly elevated the abundance of certain opportunistic pathogens in the host’s liver and lungs. This marked elevation contributes to the establishment of infection. Through cluster analysis, we found that the changes in the microbiota of the liver and lungs were independent of the microbial flora carried by *T. canis* adults. However, whether the changes are due to the migration of larvae remains to be explored. In short, *T. canis* infections have a significant impact on the abundance and diversity of flora in the host tissues, and the changes in microbiota abundance and diversity could further influence tissue homeostasis and immune responses, thus regulating the establishment of infection.

## Abstract (fr)

La toxocarose est une maladie parasitaire zoonotique largement répandue dans le monde. Les adultes de *Toxocara canis* parasitent le tractus intestinal grêle des canidés et les larves migrent vers le foie et les poumons avant d’atteindre leur destination finale. Nos expériences précédentes ont confirmé que l’infection par *T. canis* pouvait affecter la composition de la flore microbienne intestinale de l’hôte. Dans cette expérience, nous analysons plus en détail les effets potentiels de l’infection par *T. canis* sur la flore microbienne hépatique et pulmonaire de l’hôte. En utilisant le séquençage à haut débit de l’ARNr 16s, associé à diverses techniques d’analyse bioinformatique, notre étude a révélé que l’infection par *T. canis* augmentait significativement l’abondance de certains agents pathogènes opportunistes dans le foie et les poumons de l’hôte. Cette augmentation marquée contribue à l’établissement de l’infection. Grâce à une analyse de cluster, nous avons découvert que les changements dans le microbiote du foie et des poumons étaient indépendants de la flore microbienne hébergée par les adultes de *T. canis*. Il reste cependant à déterminer si ces changements sont dus à la migration des larves. En bref, les infections par *T. canis* ont un impact significatif sur l’abondance et la diversité de la flore dans les tissus de l’hôte, et les changements dans l’abondance et la diversité du microbiote pourraient influencer davantage l’homéostasie tissulaire et les réponses immunitaires, régulant ainsi l’établissement de l’infection.

## Introduction

Toxocariasis is an important zoonotic parasitic disease that is easily overlooked. In both the final and the paratenic hosts, the infectious larvae emerge from the eggs in the small intestine of the hosts, and penetrate into the intestinal wall, migrate to the liver and lungs with the blood circulation [28]. Therefore, during migration of the infectious larvae, the liver and lungs of the host are damaged [9, 20]. Our previous experiments have confirmed that *Toxocara canis* infection can regulate the composition of host intestinal microbial flora [21], and some studies have shown that the intestinal microbial flora is involved in regulating the occurrence and development of liver and lung diseases, such as chronic hepatitis B (CHB), chronic hepatitis C (CHC), pulmonary hypertension, chronic obstructive pulmonary disease (COPD), and lung cancer, through the “hepato-intestinal axis” and “lung-intestinal axis”. On the other hand, the presence of liver or lung diseases frequently correlates with gastrointestinal dysfunction. For instance, liver diseases can trigger inflammation or disrupt the normal synthesis and secretion of bile, ultimately having a downstream impact on digestion. Analogously, lung diseases often lead to reduced oxygen saturation in the bloodstream, subsequently affecting the functionality of the gastrointestinal tract [7, 13, 18, 25]. At present, researchers mainly focus on the effects of bacterial, viral, and parasitic infections on host intestinal microbial flora, but do not know whether the liver and lung damage caused by parasitic infections is related to the changes in microbial flora in these organs. In addition, *T. canis* itself also carries a variety of bacteria [21], so whether the changes in the microbial flora of the host tissue caused by the infection are related to the bacteria carried by *T. canis* is worth exploring. Therefore, this study focused on the changes in the composition, abundance, and diversity of microbial flora in the liver and lungs of hosts with *T. canis* infection and further analyzed the correlation between the microbial flora changes in the host tissues and the microbial flora carried by *T. canis*.

## Material and methods

### Ethics

The animal study protocol was reviewed and approved by the Hainan University Institutional Animal Care and Use Committee (HNUAUCC-2023-00201). All animal procedures used in this study were performed in accordance with the Guide for the Care and Use of Laboratory Animals (National Research Council, Bethesda, MD, USA) and recommendations of the Animal Research: Reporting of *In Vivo* Experiments (ARRIVE) guidelines. The dogs were housed in the individual cages with free access to a standard diet and acclimatized to the experimental environment (http://www.nc3rs.org.uk/arrive-guidelines).

### Animals

A total of 7 female Chinese rural dogs (5–6 months old, 6–7 kg, BMI: 21–23) were used for this experiment. The characteristics of the dogs used in this experiment were consistent with those in our previous studies [21].

### Experiment grouping and sampling

First, we divided dogs into a positive infected group and a negative control group through fecal examination. At the same time, negative and positive dogs were distinguished based on whether larvae were found in the intestines after dissection, and whether larvae were detected in liver and lung tissues by the Bellman method. After euthanizing and dissecting, intestinal contents (CI, II group), liver (CLi, ILi group) and lung tissues (CLu, ILu group) were collected from the dogs in the control group and the infected group. At the same time, the *T. canis* adults were collected from the intestines of the dogs in the positive infected group (Tc group), and the female adult uterus was separated to obtain the eggs (Egg group), and the intestinal tract (IT group) and genital tract (GT group) were also separated for further analysis. Sampling was performed in a sterile operating room, and during the sampling procedure, we made sure that all samples were not contaminated. After collection, each sample was put into an appropriate sterile tube, which was then snap-frozen in a liquid nitrogen tank.

### Preparation of meta amplicon library

The library was constructed employing 2 × Phanta Max Master Mix polymerase (VAZYME, Nanjing, China). Target amplification of the V3-V4 variable regions within the 16S rDNA of bacteria was achieved by using specific forward and reverse degenerate PCR primers (338F: 5′-ACTCCTACGGGAGGCAGCAG-3′, 806R: 5′-GGACTACHVGGGTWTCTAAT-3′). The PCR enrichment process was executed in a 50 μL reaction volume containing 30 ng of template DNA and fusion PCR primers. The thermal cycling protocol comprised an initial denaturation step at 95 °C for 3 min, followed by 30 cycles of denaturation at 95 °C for 15 s, annealing at 56 °C for 15 s, and extension at 72 °C for 45 s, culminating in a final extension at 72 °C for 5 min. PCR products were purified by DNA magnetic beads (BGI, LB00V60). The validated libraries were used for sequencing on an Illumina MiSeq/Hiseq platform (BGI, Shenzhen, China), following the standard pipelines of Illumina, and generating 2 × 250/300 bp paired-end reads.

### Bioinformatics analysis

The original sequencing data were processed as follows to obtain Clean Data. The specific steps were as follows: Reads matching primers were selected by cutadapt v2.6 software to remove primer and joint contamination, and fragments of the target region were obtained. Adopted the method of removing low quality by window: set 30 bp as the window length. If the average quality value of the window was lower than 20, deleted the read end sequence from the window and removed the reads whose final read length was lower than 75% of the original read length. N-containing reads were removed. Removed low-complexity reads (10 ATCG in a row) to obtain the final Clean Data. Then, the Divisive Amplicon Denoising Algorithm (DADA2) in QIIME2 was used to remove noise, and Amplicon Sequence Variants (ASVs) were obtained. The ASVs were 100% similar. The main process was as follows: Used qiime tools import to import the filtered double-ended sequence. Used the qiime DADA2 denoise-paired command to construct the feature list of the imported double-ended sequences based on the DADA2-paired method. Used qiime tools export to convert the feature list into a format that can be viewed directly. After obtaining the ASV representative sequence, RDP classifier software was used to compare the ASV representative sequence with the database for species annotation, and the confidence threshold was set to 0.6. Filtered the comment results as follows: Removed ASV without comment results. Unannotated results do not belong to the species in the analysis. The remaining ASVs can be used for later analysis. Mothur software (v.1.31.2) was used for Alpha diversity analysis, which is the analysis of species diversity in a single sample, including Chao index, Ace index, Shannon index, Simpson index, etc. QIIME (v1.80) software was used to perform Beta diversity analysis, which is used to compare the size of the difference in species diversity between samples. PICRUST2 (v2.3.0-b) (Phylogenetic Investigation of Communities by Reconstruction of Unobserved States) is a software that predicts functional abundance based on labeled gene sequences.

### Statistical analysis

The statistical analysis was conducted using SPSS software version 20.0. Hypothesis testing procedures followed a two-sided approach, providing measurements and corresponding *p* values. The threshold for determining significant differences was set at a *p* value less than 0.05. Additionally, a one-way analysis of variance (ANOVA) was used for further comparisons between groups.

## Results

### 16S rRNA high-throughput sequencing results

**Figure 1:**
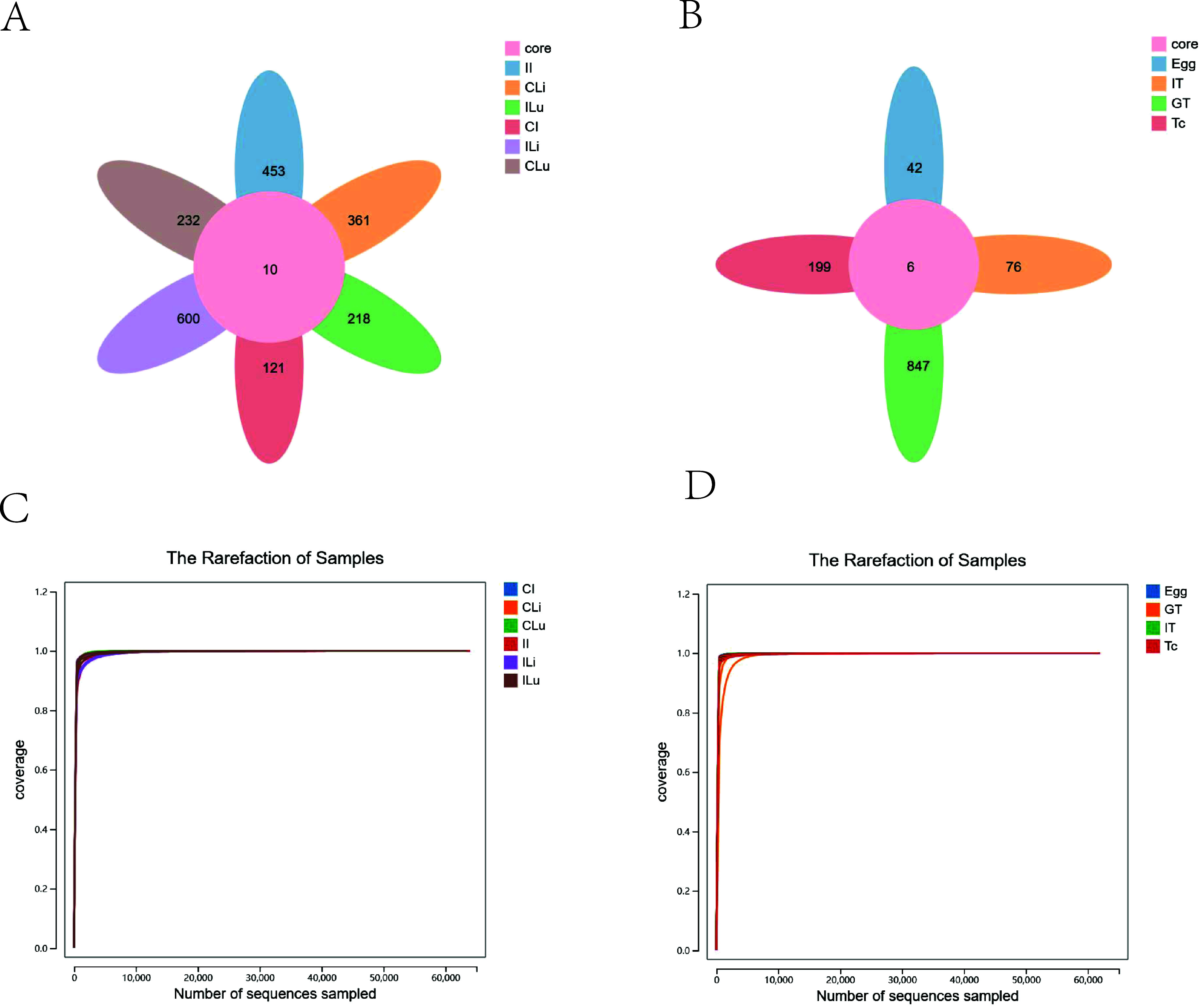
ASV statistical analysis (A–B). Core-pan ASV map of the unique and shared ASVs. The rarefaction of samples (C–D). CI, intestine samples of dogs in the control group; II, intestine samples of dogs in the *T. canis* infected group; CLi, liver samples of dogs in the control group; ILi, liver samples of dogs in the *T. canis* infected group; CLu, lung samples of dogs in the control group; ILu, lung samples of dogs in the *T. canis* infected group; Tc, *Toxocara canis* adults; Egg, egg samples of *T. canis*; GT, genital tract of *T. canis* adults; IT, intestinal tract of *T. canis* adults.

The 16S rRNA genes of tissue samples and *T. canis* samples were amplified by PCR and then subjected to high-throughput sequencing. Under the condition of 100% similarity, the reads were clustered into Amplicon Sequence Variants (ASVs). Figure 1A shows that there were significant differences in the composition of microbial flora between intestinal, liver, and lung tissues of the control and the infected groups, and the number of specific ASVs in liver tissue with *T. canis* infection (ILi group) was the highest, up to 600, while the number of specific ASVs in intestinal tissue of the control group (CI group) was the lowest, at only 121. At the same time, the number of specific ASVs in liver tissue and intestinal tissue of the infected groups were all higher than that of the control groups. Figure 1B shows that the number of ASVs in the *T. canis* genital tract tissue (GT group) was the highest, while the number of ASVs in eggs (Egg group) was the lowest. Similarly, the number of ASVs in intestinal tract tissue (IT group) was slightly higher than the number of eggs, which was significantly lower than that in adults (Tc group). Meanwhile, the rarefaction curve of each group in this study tended to be flat, indicating that the amount of sequencing data was large enough to reflect the vast majority of microbial information in the samples of all groups (Figs. 1C and 1D).

### Diversity analysis

#### Alpha diversity analysis

**Figure 2:**
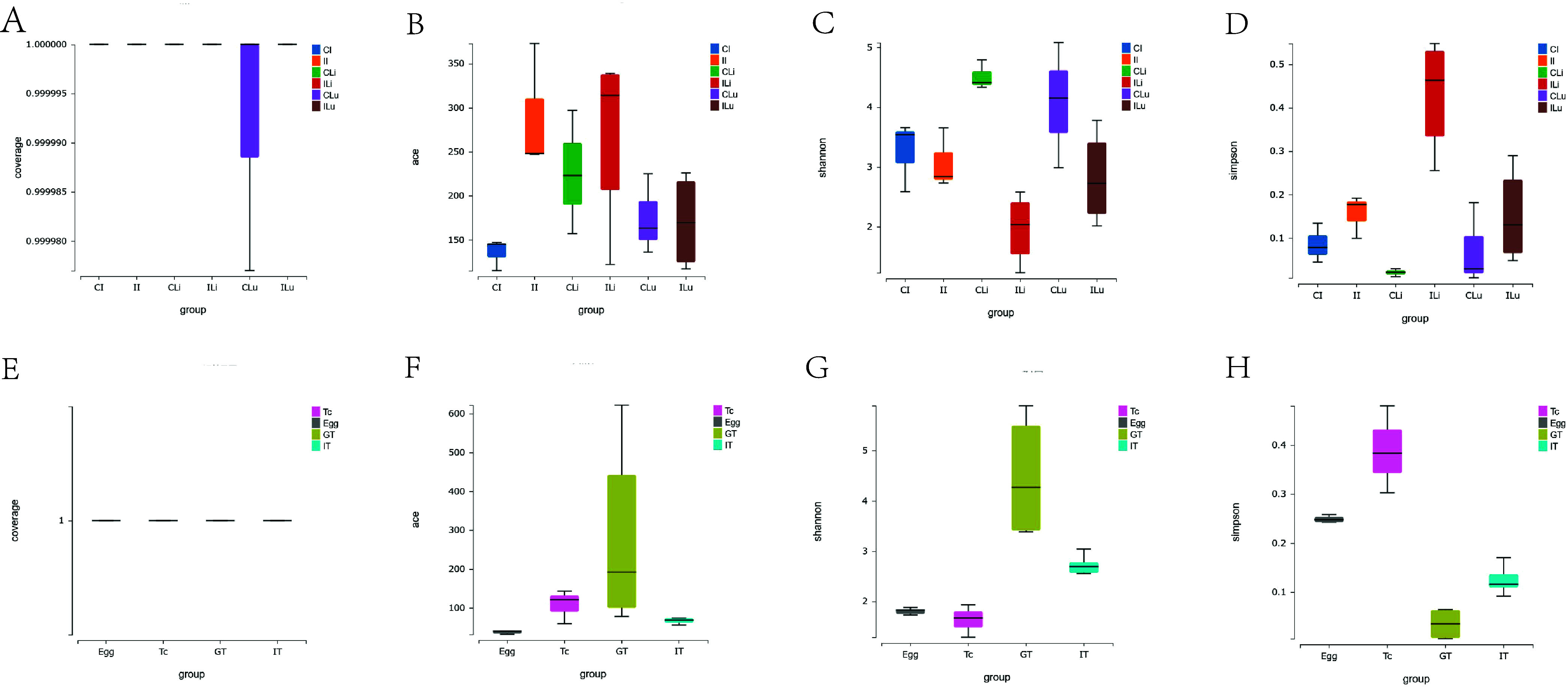
Comparison of Alpha diversity index of microbial flora. (A, E) Coverage index; (B, F) Ace index; (C, G) Shannon index; (D, H) Simpson index.

The Good’s coverage index of each group was higher than 99%, which indicates that the sequences of samples were reliable (Figs. 2A and 2E). Likewise, the Ace index showed that the richness of microbial flora in intestinal, liver, and lung tissues increased significantly after *T. canis* infection, and the flora abundance in the liver of the infected group (ILi group) was the highest, while that in the intestinal tissue of the control group (CI group) was the lowest (Fig. 2B). The Shannon index comprehensively considered the richness and evenness of the community. The higher the Shannon index, the higher the diversity of the community. The Simpson index was used to estimate microbial diversity, and the higher the value, the lower the community diversity. The Shannon index (Fig. 2C) and the Simpson index (Fig. 2D) show that the diversity of microbial flora of intestinal, liver, and lung tissues all decreased significantly after *T. canis* infection, and the flora diversity in the liver of the control group (CLi group) was the highest, while that in the liver of the infected group (ILi group) was the lowest. As can be seen from Figure 2F, there was no significant difference in the richness between the Tc, Egg, GT, and IT groups, while the diversity of microbial flora in the GT group was the highest (Figs. 2G and 2H). In general, there are certain differences in the richness and diversity of microbial flora between different tissues of *T. canis*, and there are significant regulatory effects on the richness and diversity of microbial flora in host tissues after *T. canis* infection, suggesting that *T. canis* parasitism has different effects on different host tissues.

#### Beta diversity analysis

**Figure 3:**
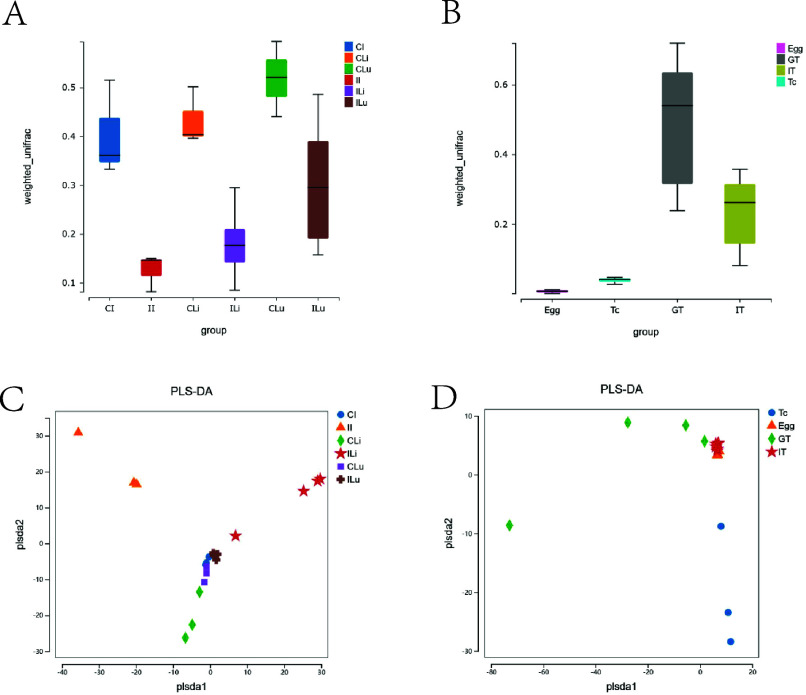
Comparison of Beta diversity index of microbial flora. (A–B) Weighted unifrac; (C–D) Partial Least-Squares Discriminant Analysis (PLS-DA).

Beta diversity indicates the comparison of composition, abundance, and phylogenetic relationships among community members. In our study, the weighted unifrac index indicated that the species composition and abundance of microbial flora in intestinal, liver, and lungs in the infected groups (II, ILi, and ILu groups) were lower than those with the control groups (CI, CLi, CLu group) (Fig. 3A). Figure 3B shows that the species composition and abundance of microbial flora in *T. canis* genital tract was the highest among all four groups (Fig. 3B). The result of Partial Least Squares Discriminant Analysis (PLS-DA) showed that the CI, II, CLi, ILi, CLu, and ILu groups formed different clusters, and the distances of the clusters of the CI, CLu, CLi, and ILu groups were closer (Fig. 3C). We also found that the diversity of microbial flora in the Egg, GT, and IT groups were similar, and the cluster is obviously different from the Tc group (Fig. 3D).

### Analysis by linear discriminant analysis effect size

**Supplementary Figure 1.**
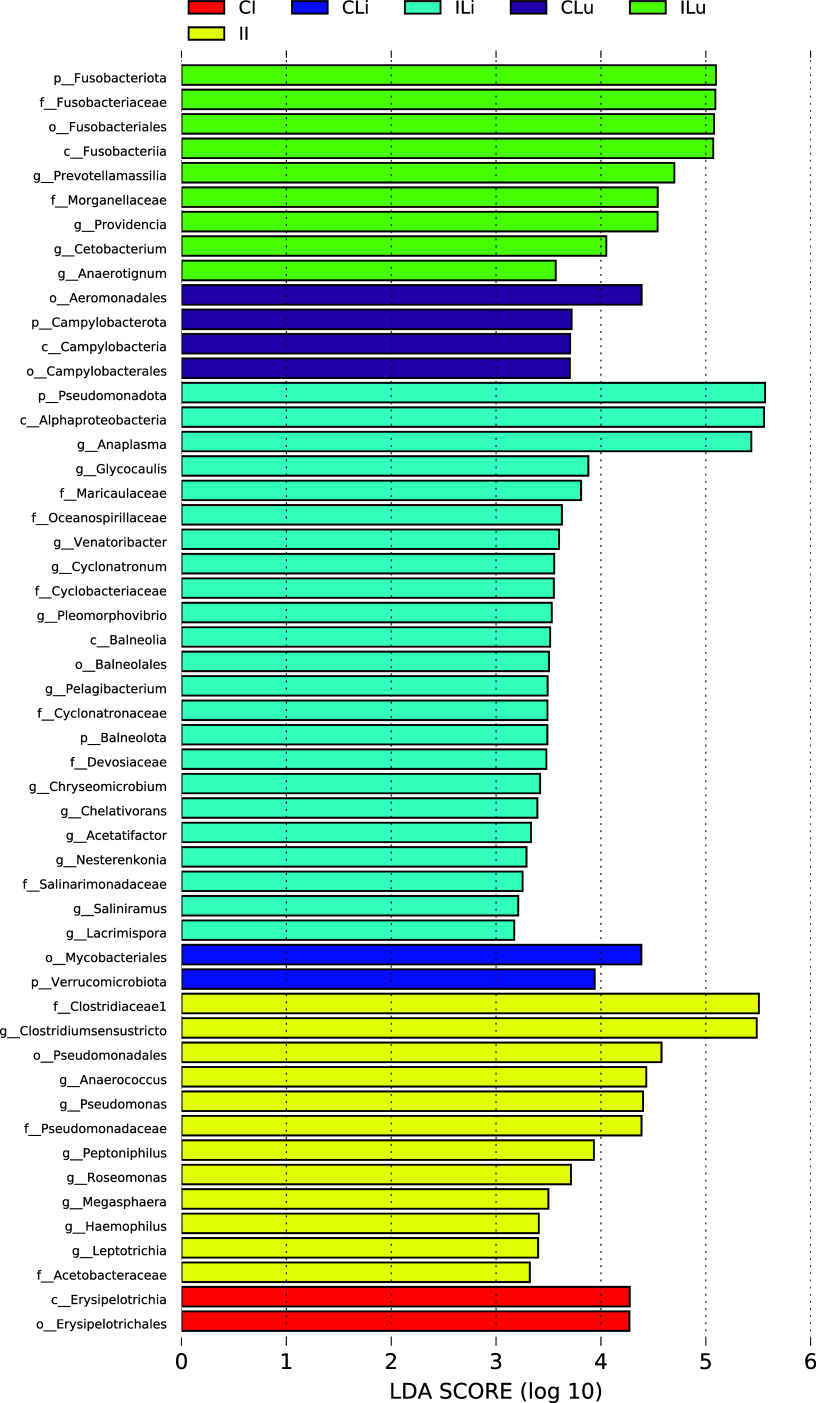
LDA value distribution histogram of the CI, II, CLi, ILi, CLu, ILu groups.

**Supplementary Figure 2.**
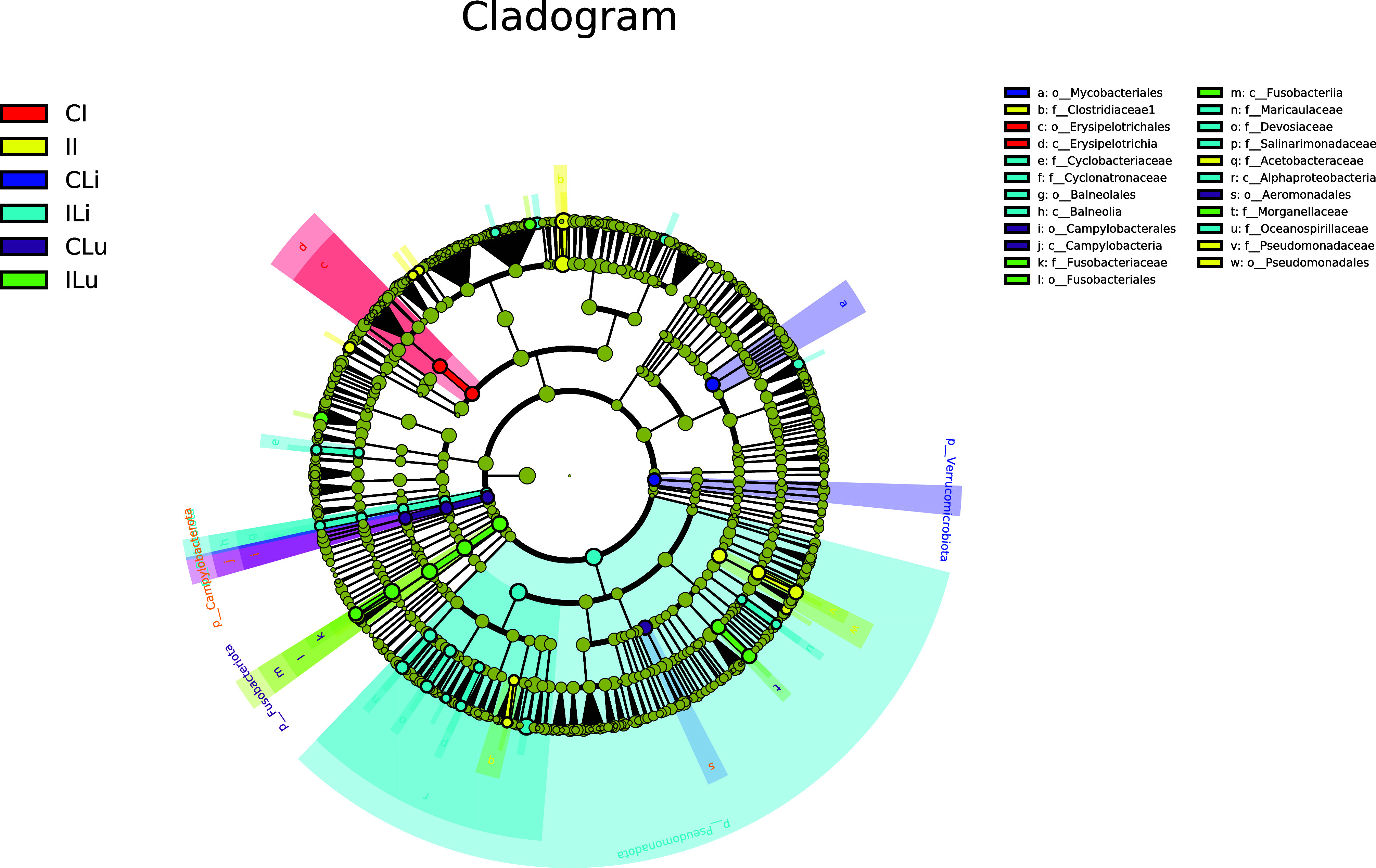
Cladogram of microbial flora of the CI, II, CLi, ILi, CLu, ILu group.

**Supplementary Figure 3.**
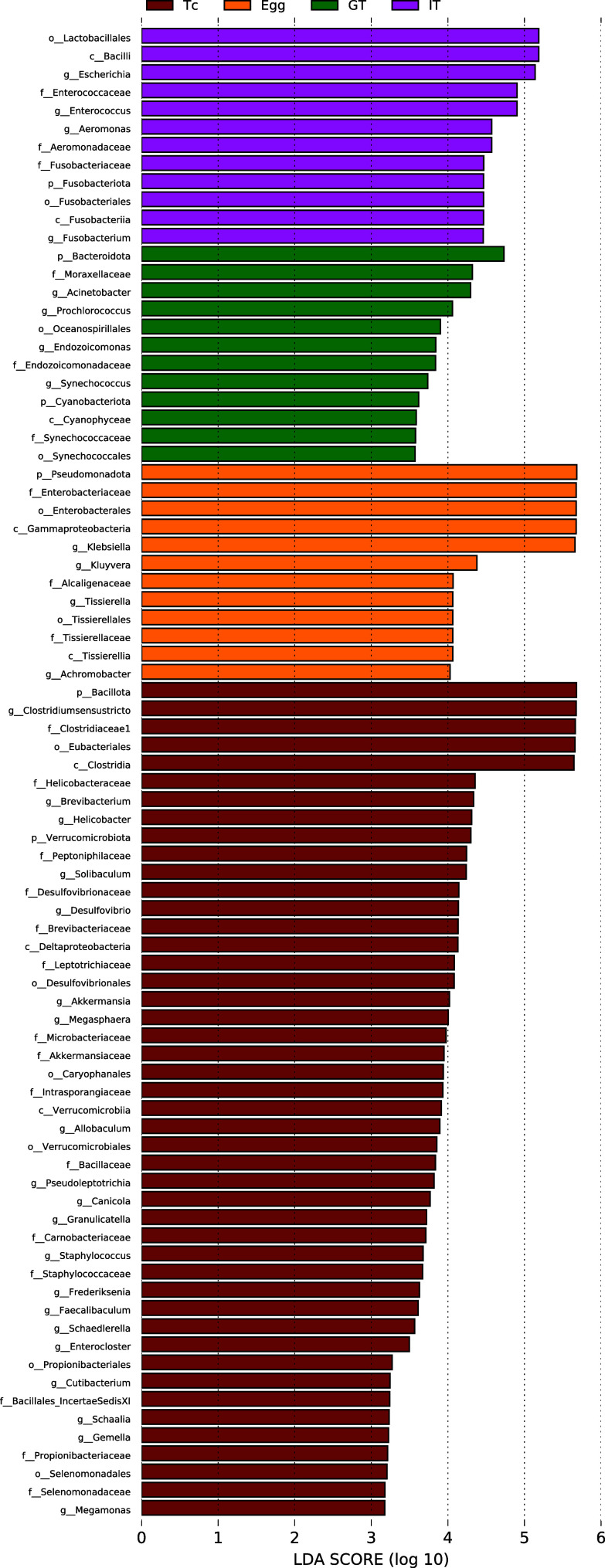
LDA value distribution histogram of the Tc, Egg, GT, and IT group.

**Supplementary Figure 4.**
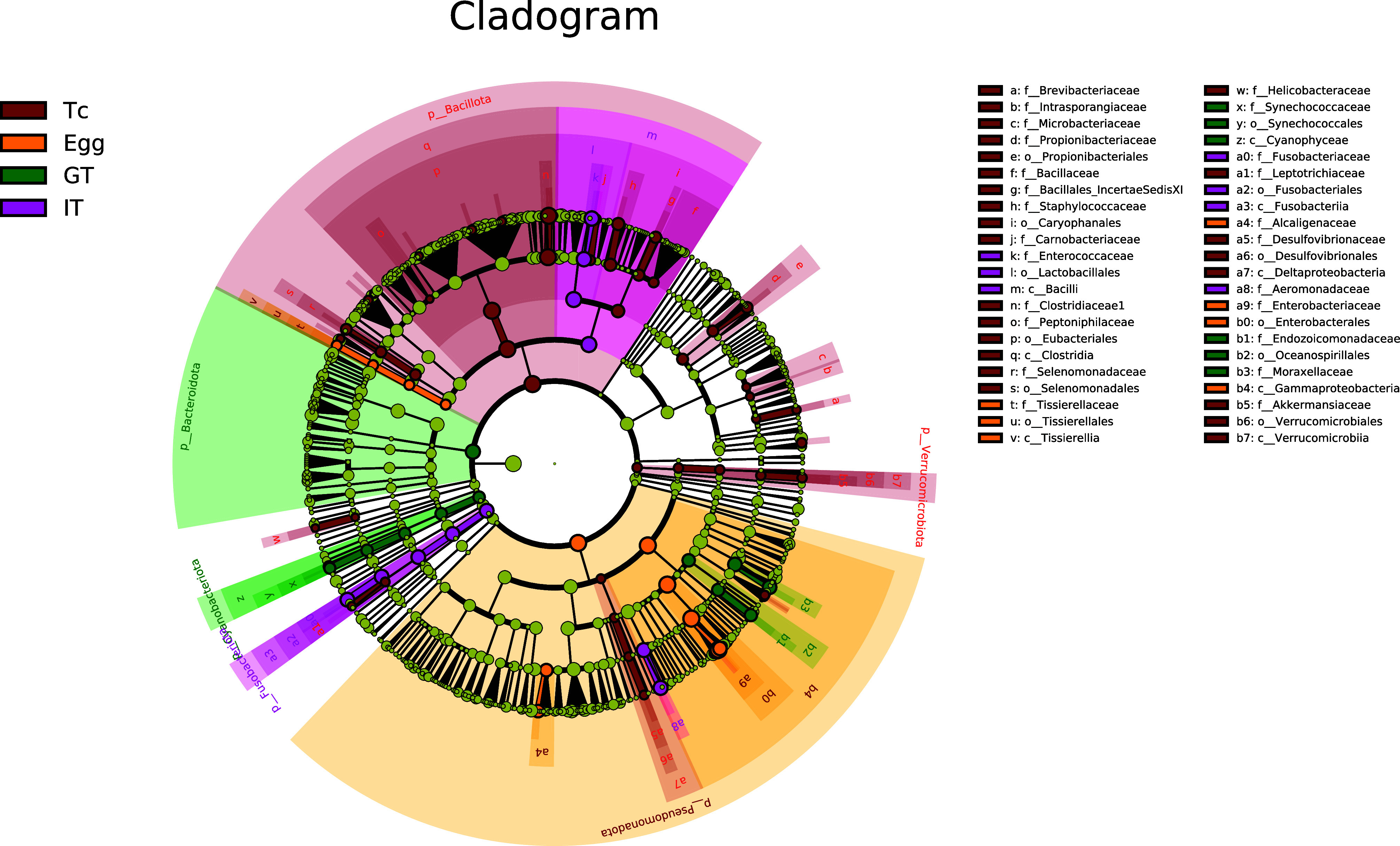
Cladogram of microbial flora of the Tc, Egg, GT, and IT group.

Linear Discriminant Analysis Effect Size (LEfSe) aided in determining the biomarkers that were significantly different and present among the different groups. As illustrated in Figure S1, LEfSe analysis of the CI, II, CLi, ILi, CLu, and ILu groups elucidated 21 biomarkers with LDA score >4. In the II group, *Clostridium sensu stricto*, and Clostridiaceae could be used as biomarkers. In the ILi group, Pseudomonadota, Alphaproteobacteria, and *Anaplasma* could be used as biomarkers. In the ILu group, Fusobacteriales, Fusobacteriota, Fusobacteria, and Fusobacteriaceae could be used as biomarkers. LEfSe clustering analysis also showed that these markers were mainly involved in phylum Pseudomonadota (see Fig. S2). As illustrated in Figure S3, LEfSe analysis of the Tc, Egg, GT, and IT groups elucidated 47 biomarkers with LDA score >4. Additionally, LEfSe clustering analysis showed that these markers were mainly involved in phylum Pseudomonadota, Bacillota and Bacteroidota (see Fig. S4).

### Species composition and difference analysis

#### Species composition and difference analysis at the phylum level

**Figure 4:**
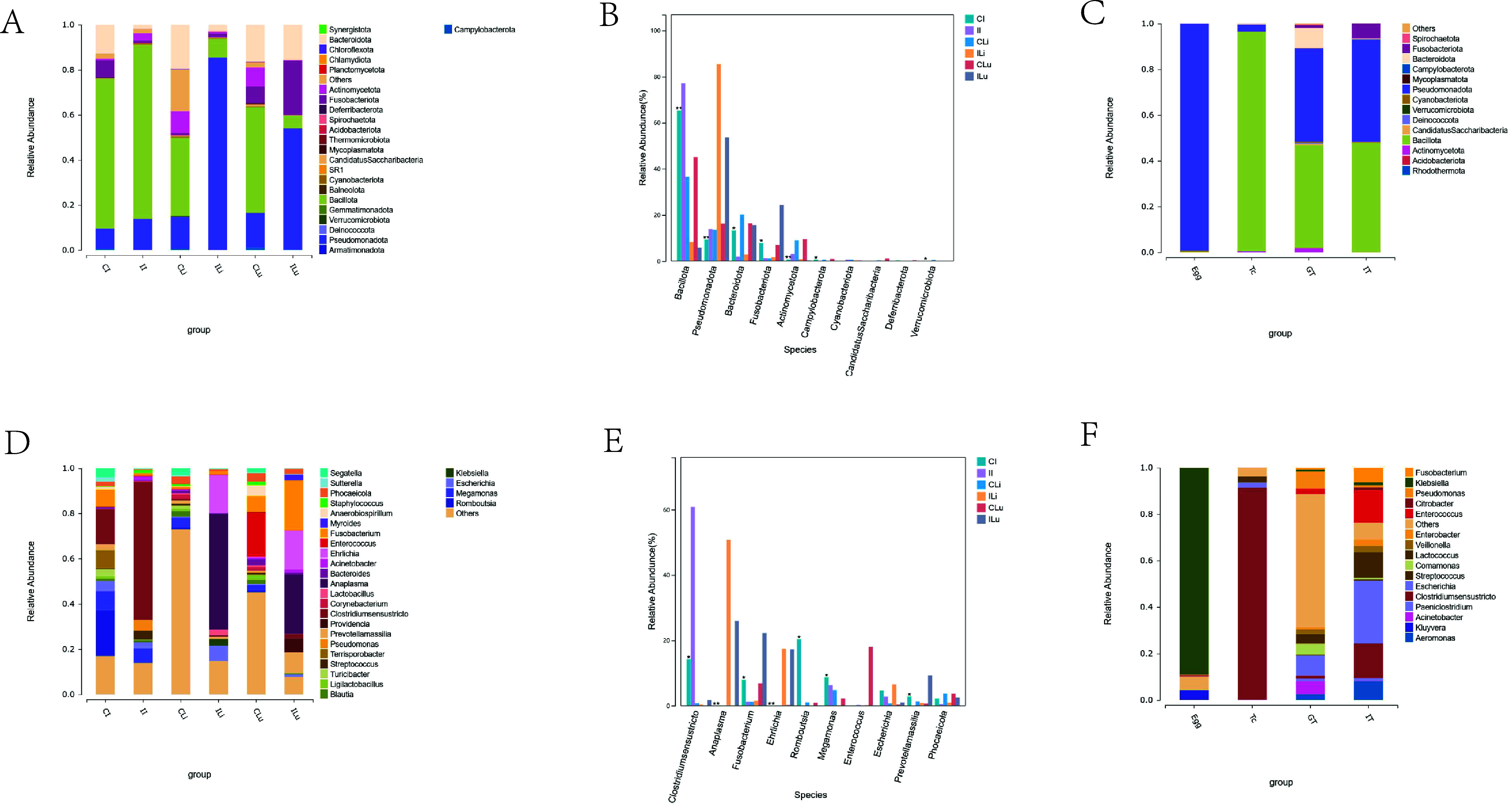
Difference in species composition of flora at the phylum level. The horizontal coordinate is the sample name and the vertical coordinate is the relative abundance of the species annotated. Species not annotated at this taxonomic level and whose abundance was less than 0.5% of the sample were combined as “Others” (A, C, D, F). The top 10 species at the phylum level in the 6 groups are showed in B and E.

As shown in Figure 4A, the species composition histogram could explain the dominant species and their relative abundance of each group. Bacillota is the dominant phylum (the value of relative abundance > 0.05) in the microbial flora of the CI, II, CLi, and CLu groups. In the ILi and ILu groups, the dominant species were from phyla Pseudomonadota. The differences in the abundance of tissue microbial flora at the phylum level among the above six groups are shown in Figure 4B. The community abundance of Bacillota in the II group was significantly higher than in the CI group, and significantly lower in the ILi and ILu groups than in the CLi and CLu groups (*p* > 0.01). The abundance of Pseudomonadota was higher in the infected groups (II, ILi, and ILu groups) compared with that in the control groups (CI, CLi, and CLu groups), and the degree of increase in the ILi and ILu groups was significantly higher than that in the II group. In addition, we found that Bacteroidota was significantly decreased in the host intestines and liver with *T. canis* infection, and the abundance in the lung had no change. As shown in Figure 4C, Bacillota was the dominant phylum in the microbial flora of the Tc group, Pseudomonadota was the dominant phylum in the microbial flora of the Egg group, and in the GT and the IT group, the dominant species were from two phyla: Bacillota and Pseudomonadota.

#### Species composition and difference analysis at the genus level

By comparing the microbial flora composition changes in intestinal, liver, and lung tissues at the genus level (Fig. 4D), it was illustrated that the dominant genus (the value of relative abundance > 0.05) in the II group was *Clostridium sensu stricto*, the dominant genera in the ILi group were *Escherichia*, *Anaplasma*, *Ehrlichia*, and the dominant genera in the ILu group were *Anaplasma*, *Prevotellamassilia*, *Ehrlichia*, *Fusobacterium*, and *Providencia*. The differences in the abundance of intestinal microbial flora at the genus level among all groups are shown in Figure 4E. The relative abundance of *Clostridium sensu stricto* in host intestinal microbial flora was increased after infection with *T. canis*. The relative abundances of *Anaplasma* and *Ehrlichia* were significantly increased in liver and lung tissues, the relative abundances of *Fusobacterium* and *Prevotellamassilia* were increased in lung tissues, and the relative abundance of *Escherichia* was increased in liver tissues after infection. As shown in Figure 4F, the dominant genus in the Tc group was *Clostridium sensu stricto*, the dominant genus in the Egg group was Klebsiella. Most of the bacteria in the GT group were not noted, and the dominant genera were *Escherichia*, *Pseudomonas*, and *Acinetobacter*. The dominant genera in the IT group were *Aeromonas*, *Lactococcus*, *Clostridium sensu stricto*, *Fusobacterium*, *Escherichia*, and *Enterococcus.*

### Function annotation via the Kyoto Encyclopedia of Genes and Genomes

**Figure 5:**
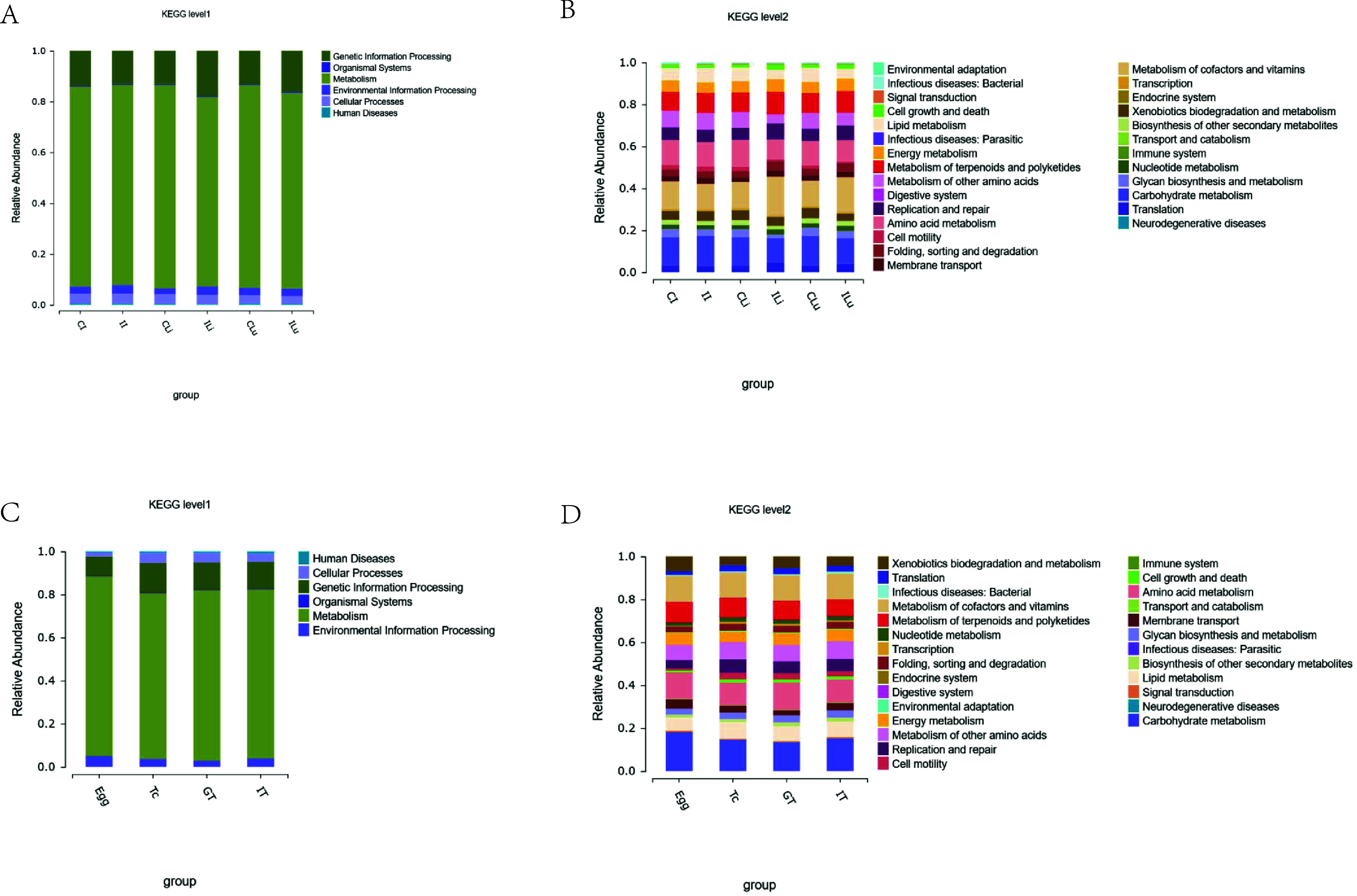
Difference in species composition of flora at the genus level. The horizontal coordinate is the sample name and the vertical coordinate is the relative abundance of the species annotated. Species not annotated at this taxonomic level and whose abundance was less than 0.5% of the sample were combined as “Others” (A, C, D, F). The top 10 species at the genus level in the 6 groups are showed in B and E.

The abundance prediction via the Kyoto Encyclopedia of Genes and Genomes (KEGG) function in microbial flora was obtained by PICRUST2. As shown in Figure 5A, the abundance of microbial flora involved in metabolic function in livers and lungs decreased after infection with *T. canis*. Further analysis showed that the abundance of bacteria involved in carbohydrate metabolism and lipid metabolism was increased in the intestinal tissue with *T. canis* infection. Meanwhile, the abundance of bacteria involved in metabolism of other amino acids and lipid metabolism was decreased in liver and lung tissues of the infected group (Ili and ILu groups), and the abundance of bacteria involved in metabolism of cofactors and vitamins was increased (Fig. 5B). The microbial flora of *T. canis* is mainly involved in metabolic processes (Fig. 5C), and the abundance of bacteria involved in carbohydrate metabolism was higher in the Egg group than that in the other three groups. In addition, the abundance of bacteria involved in lipid metabolism and replication and repair function was lower in the Egg group than that in the other groups (Fig. 5D).

### Cluster analysis

**Figure 6:**
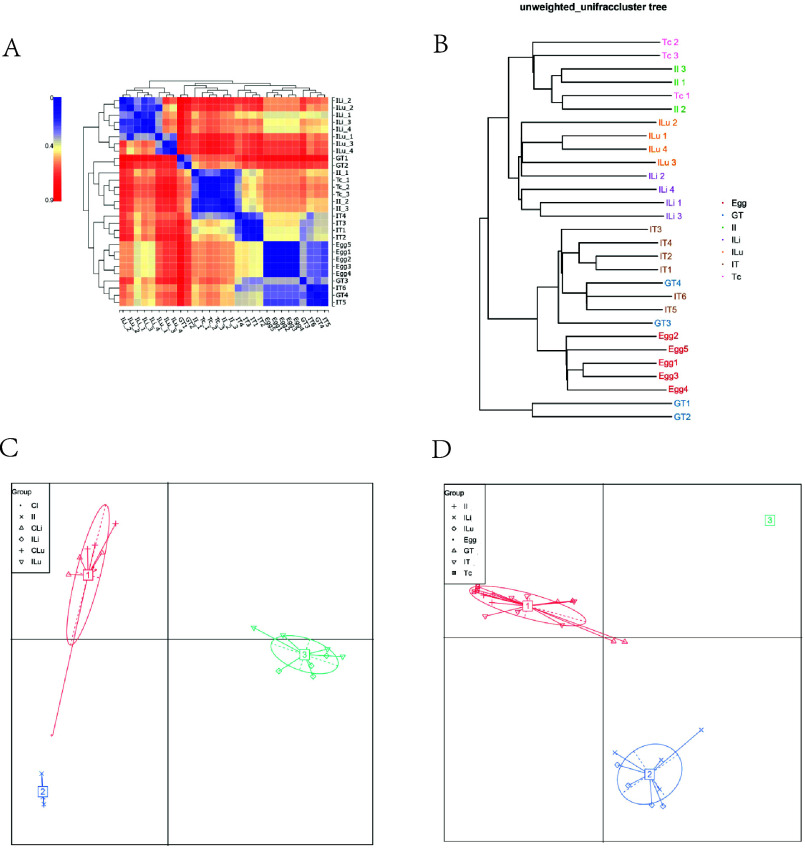
Cluster analysis. The Beta diversity matrix heatmap visualizes the Beta diversity data through graphs and clusters samples. Samples with similar beta diversity are clustered together. Darker colors indicate stronger correlation between species (A). UPGMA clustering tree (B). The shorter the branch length between samples, the more similar the two samples. The greater the distance, the greater the difference in species composition. Microflora classification analysis mainly studies the classification method of dominant microflora structure of different samples by statistical clustering methods (C, D).

In order to further analyze whether the changes in microbial flora in the host tissues after infection with *T. canis* were related to the microbial flora carried by the parasites themselves, we conducted cluster analysis of the microbial florae of each group. First, through the beta diversity heat map, we could observe that the microbial flora composition of the II group was similar to that of the Tc group; the microbial flora of the ILi group was similar to that of the ILu group. In addition, the microbial flora of the GT group was similar to that of the IT group, and there was a certain similarity between the Egg group and the above two groups (Fig. 6A). The Ultrametric Phylogenetic Matrix Analysis (UPGMA) Cluster Tree showed that the II and Tc groups were on the same branch, the ILi and ILu groups were on the same branch, and the GT, IT, and Egg groups were on the same branch (Fig. 6B). We conducted a typing analysis of the florae in each tissue of the infected group and the control group and found that the structure of dominant microflora of the CI, CLi, and CLu groups was similar, and the structure of dominant microflora of the ILi and ILu groups was similar (Fig. 6C). Then, we further analyzed whether the changes in microflora composition in tissues of the infected groups were associated with *T. canis* infection. The results showed that the structures of dominant microflora of the II, Tc, GT and IT groups were similar, and the structure of dominant microflora of the ILi group was similar to the ILu group (Fig. 6D). In general, the changes in intestinal microbial flora in the infected group were caused by the parasite, while the changes in liver and lung flora were unrelated to the adults, but the structure of dominant flora between the ILi group and the ILu group was similar, and not similar to the II group. Therefore, we speculated that part of the reason for the changes in liver and lung flora might be related to larval migration.

## Discussion

*Toxocara canis* adults inhabit the intestinal tract of their host. This infestation can cause a variety of symptoms, ranging from gastrointestinal distress and nutrient malabsorption to potential organ damage. Therefore, it is crucial to identify and treat *T. canis* infections promptly to minimize their potential impact on the host’s health [28]. *Toxocara canis* larvae can migrate to the liver and lungs to infect both definitive and paratenic hosts. The liver and intestines engage in bidirectional communication via the portal vein, bile duct, and systemic circulation, facilitating the exchange of vital substances and information between the two organs [5]. The gut-liver axis serves as the physiological foundation for the interaction between the intestinal microbial flora and the liver [30]. When the intestinal mucosal barrier function is compromised, the intestinal microbial flora and its metabolites can infiltrate the liver as antigenic signals. This triggers inflammatory responses and immune regulation within the liver [2, 27]. On the other hand, the liver exerts a reciprocal influence on the intestinal microbial flora by modulating the secretion of bile acids and their associated signalling pathways, thereby regulating the abundance and diversity of the intestinal microbiota [6, 10, 19, 26, 29]. Niza et al. have confirmed the presence of bacteria in the liver of healthy dogs [16]. In the present study, we explored the changes in the liver microbial flora when dogs are infected with *T. canis*. In addition, in order to further analyze whether the changes in liver microflora were affected by changes in intestinal microflora due to the presence of the hepatointestinal axis, we also explored the intestinal microflora of the control group and the infected group. At the same time, in order to ensure alignment with the previous experiment, the characteristics of the dogs used in this experiment were consistent with those used in the previous experiment [21]. We collected intestine and liver tissues from the infected group and the control group, and analyzed the diversity and abundance of liver microbial flora by 16s rRNA high-throughput sequencing technology combined with a series of bioinformatics analysis methods. The experimental results showed that the changes in intestinal microbial community composition and diversity of dogs in the infected group and the control group were consistent with the previous experimental results [21]. *Toxocara canis* infection induced a significant elevation in bacterial richness, while the bacterial diversity decreased in the intestines and liver. The abundance of Pseudomonadota significantly increased and that of *Bacteroidota* significantly decreased in the microbial flora of the liver and intestines, while the abundance of Bacillota significantly decreased in the liver and increased in the intestines. However, the Bacillota/Bacteroidetes ratio was increased in the liver and intestinal microbial flora of the *T. canis* infected group. It has been confirmed that Pseudomonadota is associated with inflammatory diseases [11, 14, 22, 23]. The increased abundance of Pseudomonadota or *Enterococcus* and the decreased abundance of butyrate-producing bacteria, such as Bacteroidetes and Bacillota, in the gastrointestinal tract are associated with heightened risks of infection. At the genus level, we found significant increases in the abundance of some pathogenic and opportunistic pathogenic bacteria, such as *Anaplasma*, *Ehrlichia*, or *Escherichia*, in the liver microbial flora after *T. canis* infection. In general, *T. canis* infection can cause a significant increase in Bacillota in the host intestines, which played a role in inhibiting the intestinal inflammatory response and was conducive to its long-term survival [21]. On the other hand, *T. canis* infection mainly increased the proportion of some pathogenic bacteria in the host liver, which had an adverse effect on the host and was conducive to the establishment of infection.

Similarly, intestinal microbial flora may be involved in regulating the occurrence and development of lung diseases through the lung-intestinal axis [13]. During the migration, *T. canis* migrates to the lungs, and researchers have found hepatopulmonary migration in the early stage of infection [24]. Therefore, we speculated that *T. canis* infection could also affect the host lung microbial flora. Researchers have demonstrated that the lung microbiota possesses a unique characteristic and is different from the microbiota of the intestinal tract in healthy individuals, both in terms of high-level taxonomy (phylum) and functionality (KEGG module) [4]. In healthy adults, the lung microbiota is primarily composed of two bacterial phyla: Bacteroidetes and Bacillota [8, 13]. Lindsey and Pierce confirmed the presence of bacteria in the lungs of dogs [12]. In our study, we found that the abundance of Pseudomonadota in the lung microbial flora is significantly increased, and the abundance of Bacteroidetes significantly decreased, which was similar to the changes in the liver microbial flora. However, *T. canis* infection had no significant effect on the abundance of Bacillota in the lungs. In addition, a significant increase in the abundance of Fusobacteriota was detected in the lungs of the infected group. Fusobacteriota can elicit host proinflammatory responses [15, 17]. At the genus level, some pathogenic bacteria, such as *Anaplasma*, *Ehrlichia*, and *Providencia* were also increased in the lung microbial flora of the infected group. In general, the effects of *T. canis* infection on the lung microflora were similar to the changes in liver microflora, and the infection leads to a significant increase in the proportion of bacteria involved in inflammation and some pathogenic bacteria in the liver and lungs.

Based on our results, we confirmed that *T. canis* parasitism played a significant regulatory role in the composition of host intestinal, liver, and lung microbial florae. We speculated that the influence of *T. canis* infection on host intestinal microbial flora was caused by adults [21]. In order to verify the hypothesis, we further analyzed the microbial flora of *T. canis* adults, their genital tract, intestinal tract, and eggs. The dominant flora of the adults was Bacillota, the dominant flora of the eggs was Pseudomonadota, and the dominant florae of the genital tract and intestinal tract of *T. canis* adults were Bacillota and Pseudomonadota. By developing unique metabolic pathways and survival strategies, the bacteria in Pseudomonadota show strong environmental adaptability and can survive under various extreme conditions, such as high or low temperature, high salt, low oxygen, and others [3]. Pseudomonadota can decompose harmful substances in the soil, which is conducive to the survival and development of eggs in the soil. Bacillota bacteria are able to break down complex carbohydrates and produce short-chain fatty acids that provide energy. In addition, Bacillota can produce antibacterial substances and inhibit the growth of other pathogens, maintaining the microbial ecological balance [1]. Thus, the higher abundance of Bacillota carried by *T. canis* adults was conducive to their long-term survival in the host intestines by maintaining intestinal homeostasis. Meanwhile, the dominant Pseudomonadota and Bacillota bacteria in the intestinal tract of adults mainly participate in metabolic processes and provide energy for *T. canis* development. Similarly, the functions of Pseudomonadota and Bacillota in the genital tract of the parasite may mainly maintain the homeostasis of the internal environment.

Since the abundance of Pseudomonadota and Bacillota in the host tissues was also changed significantly, we hypothesized that changes in the microflora in the tissues were closely related to *T. canis* infection. Next, through cluster analysis, we found that the intestinal, liver and lung microbial florae of the control group were in the same cluster, and the microbial florae of the liver and lungs in the infected group were in the same cluster, but not in the same cluster as the intestinal microbial flora in the infected group. Then, we included microbial flora of *T. canis* for cluster analysis. It was found that the intestinal microbial flora of the host infected with *T. canis* was in the same cluster as the microbial flora of *T. canis* adults, and the microbial florae of the genital tract, intestinal tract, and eggs of *T. canis* were in the same cluster, but were not in the same cluster as the liver and lung microbial florae in the infected group. Therefore, we believe that the changes in the host intestinal microbial flora after *T. canis* infection were caused by the flora carried by the adults to a certain extent, and the flora changes are conducive to its long-term survival in the host [21]. The changes of liver and lung microbial florae after *T. canis* infection were not related to adults and eggs, and were not caused by the changes in intestinal microbial flora. More importantly, the liver and lung microbial florae in the infected group were clustered together, and we speculate that the changes in liver and lung microbial flora might be due to larval migration. However, the liver and lung microbial florae in the control group were also clustered, so whether the changes in the liver and lung microbial florae were indirectly caused by larvae parasitism or directly caused by the microbial flora carried by larvae remains to be further explored. In addition, these current results could not determine the relationship between infection and microbial flora changes. For example, whether infection with *T. canis* leads to the change of host flora composition or the change of host flora leads to the enhancement of *T. canis* infection remains to be clarified through further exploration.

## Conclusion

*Toxocara canis* infection causes significant changes in the abundance and diversity of microbial florae in the host’s intestines, liver, and lungs. *Toxocara canis* adult parasitism can lead to the abundance of Bacillota being significantly increased in the host intestines, thus playing an anti-inflammatory function conducive to its long-term survival in the intestines. In addition, the increased Bacillota is likely to come from the microbial flora carried by the *T. canis* adults. The diversity of microbial florae in the host liver and lungs also changed after *T. canis* infection, and the abundance of some inflammation-related bacteria, such as Pseudomonadota, and pathogenic bacteria, such as *Anaplasma* and *Ehrlichia*, increased significantly, which may be conducive to the establishment of infection in liver and lung tissues. In addition, the changes in microbial flora in the liver and lungs of the infected group were similar, and cluster analysis showed that they are in the same cluster. The changes in microbial flora in the liver and lungs were shown to be independent of *T. canis* adults and eggs, and have low association with intestinal microbial flora. However, whether the changes in microbial flora in the liver and lungs are caused by migration of larvae remains to be further explored.

## Data Availability

The raw sequencing data were deposited in the SRA database as PRJNA1219040.

## Supplementary material

The supplementary material for this article can be found at https://www.parasite-journal.org/10.1051/parasite/2025011/olm.
